# Knowledge and Perceptions of Migraine Management and Trigger Factors Among Medical Students in Riyadh, Saudi Arabia: A Cross-Sectional Study

**DOI:** 10.7759/cureus.75666

**Published:** 2024-12-13

**Authors:** Samer Alzahrani, Moyser Z Mulla, Adnan Z Mulla, Ibrahim T Almegbil, Sultan K Alblaihi, Osama M Alkhani, Fawaz F Alzaben

**Affiliations:** 1 Department of Family Medicine, Imam Mohammad Ibn Saud Islamic University (IMSIU), Riyadh, SAU; 2 Department of Family Medicine, Primary Health Care Corporation, Riyadh, SAU; 3 College of Medicine, Imam Mohammad Ibn Saud Islamic University (IMSIU), Riyadh, SAU; 4 Department of Research, Imam Mohammad Ibn Saud Islamic University (IMSIU), Riyadh, SAU

**Keywords:** kingdom of saudi arabia (ksa), medical students, migraine, perceptions, riyadh city, trigger factors

## Abstract

Introduction: Migraines pose a significant risk to the health of individuals who suffer from them. Consequently, healthcare specialists must have sufficient knowledge and awareness to recognize and treat migraines. This study aimed to assess medical students' knowledge and perception of migraine-triggering factors and management methods.

Methods: This cross-sectional study was conducted at multiple medical school universities in Riyadh, Saudi Arabia. An online survey approach was employed.

Results: In total, 233 participants (77% response rate) filled out the survey. Male students were predominantly represented (81%), and most students were in their fifth year (32.2%) or first year (27%) of medical school. Most participants (60.5%) considered that the prevalent age at presentation of migraines was between 17 and 30 years. Knowledge of treatment modalities compared with the year of study was significantly reported to be medication (53.4%), rest (41%), and alternative therapies, such as acupuncture (4.4%), for managing migraines (p ≤ 0.001). The most commonly recognized triggering factors were light (90.6%), stress (86.7%), and inadequate sleep (85.4%). Statistically significant recognized risk and trigger factors among students with different years of medical school training were female gender (p ≤ 0.001), certain odors (p = 0.018), and hormonal changes (p = 0.043).

Conclusion: This study demonstrated the knowledge and perceptions of medical students with different training levels regarding identifying migraine triggers and the required treatments. Although the findings indicated that students demonstrated a general knowledge and recognition of migraine triggers and treatments, an in-depth study on managing individual triggering factors is necessary.

## Introduction

Migraines are a type of complex neurological headache disorder. They can manifest as headaches that are often unilateral and throbbing and may be episodic or chronic in nature [1]. Accurate identification and management of patients presenting with migraine symptoms pose a challenge to healthcare systems [2].

The International Classification of Headache Disorders 3 (ICHD-3) has set the diagnostic criteria for migraines without aura: "headache that lasts from 4-72 hours, unilateral location, pulsation nature, moderate to severe intensity, and aggravated by routine physical activity’. Two of these features need to be present for the diagnosis to be made [3].

In 2021, 3.1 billion cases of migraines were reported globally [4-6]. In the United States, the age-adjusted prevalence of migraines was 15.9% in 2018 [7]. In Saudi Arabia, the prevalence is as high as 37% of the studied population [8,9]. Migraines were also considered the second largest global contributor, at 16.3%, to disability-adjusted life years in 2016 and were responsible for approximately 5% of the years lived with disability in 2023 [6,10]. Migraine prevalence also appears to be higher in women than in men. However, a variation in peak presentation may occur at preadolescent ages, during which the prevalence is higher in boys than in girls [4,5].

The pathophysiology of migraines has yet to be established. One theory proposes the involvement of both the central and peripheral nervous systems via the trigeminal sensory ganglion and large meningeal artery. Other theories hypothesize the involvement of meningeal and dural inflammation in the brain [1,2].

The factors that trigger or aggravate migraines may vary. Studies have noted that people who suffer from migraines report emotional stress, insufficient sleep, and certain odors as triggers [11]. Some studies have classified triggers into behavioral (e.g., stress, mental overload, and irregular sleep), environmental (e.g., loud noises, light, and odors), dietary (e.g., caffeine, nitrates, and alcohol), and pharmacological types [12]. An online article published by the American Migraine Foundation identifies 10 triggers, namely stress, changes in sleep patterns, hormones, caffeine and alcohol consumption, changes in weather, diet, dehydration, light, smells, and medication overuse. It provides advice on how to deal with each trigger [13].

It is evident from past research that a complex array of elements and interactions between them initiate migraines. Considering the multifaceted nature of migraine triggers, it is imperative to rely on multiple factors for migraine management. Besides managing triggers individually, the mainstay management of migraines involves the use of analgesic medications. Eigenbrodt et al., in their comprehensive medical guide on how to treat migraine pharmacologically, discussed in depth the importance of being able to accurately identify migraines and described the availability of different first-line, second-line, and preventative therapy medication options [14]. Emerging evidence also supports the role of alternative therapies, rather than medications, in managing migraines. These alternatives can include acupuncture, physiotherapy, and even cognitive behavioral therapy; however, evidence of their success is still lacking [15].

Effective migraine management also relies heavily on training the healthcare practitioners who treat patients suffering from migraines. This management is provided at various levels of healthcare, from primary to secondary and tertiary care [14]. Therefore, healthcare practitioners, including medical students, must have good knowledge of how to diagnose migraines, recognize migraine triggers, and manage migraines. Unfortunately, past studies have shown a lack of knowledge among physicians and medical students regarding migraine diagnosis and the guidelines to follow for migraine management [16-18].

Several studies have assessed knowledge and perception regarding migraines and their triggers in the Saudi Arabian context; however, these studies have focused on the general Saudi population [19-21]. To the best of our knowledge, no studies have examined the level of knowledge of migraine triggers and management among Saudi medical students. Therefore, this study aimed to determine the extent of knowledge of migraine triggers and management options among medical students with different training levels in Riyadh, Saudi Arabia.

## Materials and methods

Study design and setting

This cross-sectional study employed an online survey approach. The survey was distributed among medical students attending the following major medical universities in Riyadh: Imam Mohammed bin Saud Islamic University, King Saud University, Almareafa University, Alfaisal University, King Saud bin Abdulaziz University for Health Sciences, and Princess Nora University. This study took place from 20 March 2024 to 30 September 2024.

Sample size

The general medical population at these medical university locations has not been reported publicly. Therefore, a general estimate was around 10,000 students during the year 2024. The sample size was calculated to be 300 participants using Raosoft (Raosoft Inc., Seattle, WA, USA) [22]. The following equation was used:

\[ n = \frac{(Z_{0.95})^2 \cdot p \cdot (1 - p)}{(0.05)^2} \]

where n is the minimum sample size, Z_0.95 is a constant (1.96), and p is the proportion or prevalence that meets our criteria. The confidence level was 95%, the precision was ± 0.05, and the margin error was 6.92%.

Participants

The convenience sampling method was employed for participant selection by inviting medical students from selected medical colleges and universities to fill out an online survey. The survey was distributed via email and various social media platforms to reach as many students as possible. Students ranging from those in their first year at a given medical school to those in training internships were invited to participate.

Instrument

The survey consisted of four main parts. The first part covered the sociodemographic details of the participants and asked specifically about their gender and their present year of medical school. The second part asked about their knowledge of migraines, particularly regarding which age groups (<16 years, 17-30 years, 31-45 years, and >45 years) and gender were most likely to present with migraines.

The third section asked students about their knowledge of various management options for treating migraines. The options provided were rest time, medication, alternative therapies (e.g., cupping or acupuncture), and some form of physical exercise.

The fourth part asked students to use a 5-point Likert scale to indicate how strongly they agreed or disagreed about various reported factors being triggers for migraines. The factors included gender (with females being more at risk), bright lights, loud sounds, certain odors, stress, physical exertion, changes in weather and atmospheric pressure, hormonal and menstrual changes, inadequate sleep, exposure to chemicals (e.g., nitrates and monosodium glutamate (MSG) found in fast food and processed meats), excessive caffeine intake, and starvation. The fourth section’s internal reliability, calculated by testing a pilot participant sample and using Cronbach’s alpha, was determined to be 0.704.

Statistical analysis

SPSS Statistics version 27 (IBM Corp. Released 2020. IBM SPSS Statistics for Windows, Version 27.0. Armonk, NY: IBM Corp.) was used to analyze the data. Category variables were summarized using frequencies and percentages. The chi-squared test (χ2) assessed the association between qualitative data reported as numbers and percentages. A p-value of less than 0.05 was considered to indicate statistical significance. During the analysis, the 5-point Likert scale was converted to a 3-point scale to illustrate agreement, disagreement, and uncertainty. This was done by combining the "strongly agree" and "agree" answers into one category and the "strongly disagree" and "disagree" answers into another category.

Ethical considerations

Ethical approval was obtained from the Imam Mohammed bin Saud Islamic University Institutional Review Board, with registration number HAPO-01-R-0011 and project number 610/2024. Informed consent was obtained from all participants after assurance of full confidentiality and anonymity.

## Results

In total, 233 participants completed the survey (77% response rate). Table 1 shows the general characteristics of the study participants. Most of the participants were male (81%). Students in their fifth year of medical school represented the most (32.3%), followed by first-year students (27%). The least represented student groups were students in their fourth year (7.7%) and those in internship training (2.1%).

**Table 1:** General sociodemographic characteristics of participants

Characteristics of participants			Number (%)
Gender		Male	189 (81.1%)
		Female	44 (18.9%)
		Total	233 (100.0%)
Year of study	1st		63 (27.0%)
	2nd		36 (15.5%)
	3rd		36 (15.5%)
	4th		18 (7.7%)
	5th		75 (32.2%)
	Intern		5 (2.1%)
	Total		233 (100.0%)

The migraine management options most frequently recognized by the participants were medications (49.8%) and rest (41%), whereas alternative therapies, such as cupping and acupuncture, were recognized by only 4.3% of the participants (Table 2). Most participants (60.5%) reported that the age at presentation of migraines was between 17 and 30 years, and the least reported age (2.1%) at presentation was less than 16 years (Table 2).

**Table 2:** Reported management modalities for migraines and reported most common age to suffering from migraines

Reported management and age		Number (%)
Management modalities	Rest for some time	96 (41.2%)
	Medications	116 (49.8%)
	Routine physical activities	11 (4.7%)
	Alternative medicine (cupping therapy, acupuncture)	10 (4.3%)
	Total	233 (100.0%)
Age to suffer from migraines	<16 years	5 (2.1%)
	17-30 years	141 (60.5%)
	31-45 years	68 (29.2%)
	>45 years	19 (8.2%)
	Total	233 (100%)

Table 3 illustrates migraine triggers and risk factors. The risk factors most recognized by the participants were light (90.6%), stress (86.7%), inadequate sleep (85.4%), and loud sounds (74.2%). This was followed by gender differences (67.4%), hormonal influences (67.4%), caffeine intake (67.4%), and certain odors (62.7%). The less-recognized risk factors included starvation (56.2%), changes in atmospheric pressure (55.8%), physical exertion (52.8%), certain foods (50.6%), and chemicals such as MSG (45.1%).

**Table 3:** Triggers and risk factors that exacerbate migraines F: female, M: male, MSG: monosodium glutamate

Trigger	Degree of agreement	Number (%)
Gender difference F>M	Agree	157 (67.4%)­
	Disagree	17 (7.3%)
	Uncertain	56 (24.0%)
Light	Agree	211 (90.6%)
	Disagree	1 (0.4%)
	Uncertain	21 (9.0%)
Loud sounds	Agree	173 (74.2%)
	Disagree	15 (6.4%)
	Uncertain	45 (19.3%)
Certain odors	Agree	146 (62.7%)
	Disagree	21 (9.0%)
	Uncertain	66 (28.3%)
Stress	Agree	202 (86.7%)
	Disagree	12 (5.2%)
	Uncertain	19 (8.2%)
Physical exertion	Agree	123 (52.8%)
	Disagree	41 (17.6%)
	Uncertain	69 (29.6%)
Weather	Agree	118 (50.6%)
	Disagree	30 (12.9%)
	Uncertain	85 (36.5%)
Changes in atmospheric pressure	Agree	130 (55.8%)
	Disagree	24 (10.3%)
	Uncertain	79 (33.9%)
Hormonal changes	Agree	157 (67.4%)
	Disagree	14 (6.0%)
	Uncertain	62 (26.6%)
Inadequate sleep	Agree	199 (85.4%)
	Disagree	12 (5.2%)
	Uncertain	22 (9.4%)
Foods and chemicals like MSG	Agree	105 (45.1%)
	Disagree	27 (11.6%)
	Uncertain	101 (43.3%)
High caffeine intake	Agree	157 (67.4%)
	Disagree	27 (11.6%)
	Uncertain	49 (21.0%)
Starvation	Agree	131 (56.2%)
	Disagree	28 (12.0%)
	Uncertain	74 (31.8%)

The highest rates of uncertainty regarding risk factors, as indicated by participants who chose the neither agree nor disagree option, were for food (43.3%), weather (36.5%), changes in atmospheric pressure (33.9%), and starvation (31.80%). The participants were also uncertain about the effects of certain odors (28.3%), hormones (26.3%), gender influence (24.0%), caffeine intake (21.0%), and loud sounds (19.3%).

Secondary analysis (Table 4) was conducted using chi-square tests between the year of the study variable (indicating the level of medical school training) and the recognized migraine triggers and risk factors. Statistically significant results were evident in the recognition of the risk factor of gender differences, with female patients presenting with migraines more often than male patients. Fifth-year medical students ranked the highest (27%) in recognizing this difference, followed by first-year and third-year students, who both scored 11.7% (p ≥ 0.001). Differences in the recognition of certain odors as a trigger for migraines were also statistically significant, with the highest reported recognition among fifth-year medical students (24.5%), followed by first-year medical students (21.5%) (p ≥ 0.018). Statistically significant differences were also evident in the recognition of changes in hormonal status as a trigger for migraines, with the highest rate of recognition demonstrated by fifth-year medical students (24.9%), followed by first-year medical students (14.2%) (p ≤ 0.043).

**Table 4:** Chi-square analysis between participant year of study, recognized triggers, and risk factors for migraines Chi-square analysis. * significance <0.05 , ** significance <0.001, results are displayed as number (%). F: female, M: male, MSG: monosodium glutamate

Trigger	Year	1st	2nd	3rd	4th	5th	Intern	Chi-Sq value	p-value
Gender difference F>M	Agree	27 (11.7%)	24 (10.4%)	27 (11.7%)	13 (5.7%)	62 (27.0%)	4 (1.7%)	34.96	<0.001**
	Disagree	11 (4.8%)	0 (0.0%)	1 (0.4%)	1(0.4%)	3 (1.3%)	1 (0.4%)		
	Uncertain	23(10.0%)	12 (5.2%	8 (3.5%)	4 (1.7%)	9 (3.9%)	0 (0.0%)		
	Total	61 (26.5%)	36 (15.7%)	36 (15.7%)	18 (7.8%)	74 (32.2%)	5 (2.2%)		
Light	Agree	54 (23.2%)	30 (12.9%)	35 (15.0%)	17 (7.3%)	70 (30.0%)	5 (2.1%)	10.44	0.403
	Disagree	0 (0.0%)	0 (0.0%)	0 (0.0%)	0 (0.0%)	1 (0.4%)	0(0.0%)		
	Uncertain	9 (3.9%)	6 (2.6%)	1 (0.4%)	1 (0.4%)	4 (1.4%)	0 (0.0%)		
	Total	63 (27%)	36 (15.5%)	36 (15.5%)	18 (7.7%)	75 (32.2%)	5 (2.1%)		
Sounds	Agree	50 (21.5%)	25 (10.7%)	25 (10.7%)	14 (6%)	55 (23.6%)	4 (1.7%)	10.10	0.431
	Disagree	1 (0.4%)	1 (0.4%)	3 (1.3%)	1 (0.4%)	9 (3.9%)	0 (0.0%)		
	Uncertain	12 (5.2%)	10 (4.3%)	8 (3.4%)	3 (1.3%)	11 (4.7%)	1 (0.4%)		
	Total	63 (27%)	36 (15.5%)	36 (15.5%)	18 (7.7%)	75 (32.2%)	5 (2.1%)		
Odor	Agree	31 (13.3%)	16 (6.9%)	24 (10.3%)	14 (6%)	57 (24.5%)	4 (1.7%)	21.43	0.018*
	Disagree	8 (3.4%)	5 (2.1%)	5 (2.1%)	0 (0.0%)	3 (1.3%)	0 (0.0%)		
	Uncertain	24 (10.3%)	7 (3%)	7 (3%)	4 (1.7%)	15 (6.4)	1 (1.4%)		
	Total	63 (27%)	36 (15.5%)	36 (15.5%)	18 (7.7%)	75 (32.2%)	5 (2.1%)		
Stress	Agree	56 (24%)	25 (10.7%)	31 (13.3%)	15 (6.4%)	71 (30.5%)	4 (1.7%)	16.45	0.087
	Disagree	2 (0.9%)	4 (1.7%)	3 (1.3%)	1 (0.4%)	2 (0.9%)	0 (0.0%)		
	Uncertain	5 (2.1%)	7 (3%)	2 (0.9%)	2 (0.9%)	2 (0.9%)	1 (0.4%)		
	Total	63 (27%)	36 (15.5%)	36 (15.5%)	18 (7.7%)	75 (32.2%)	5 (2.1%)		
Physical excretion	Agree	26 (11.2%)	21 (9%)	23 (9.9%)	12 (5.2%)	38 (16.3%)	3 (1.3%)	12.78	0.236
	Disagree	14 (6%)	6 (2.6%)	8 (3.4%)	0 (0.0%)	12 (5.2%)	1 (0.4%)		
	Uncertain	23 (9.9%)	9 (3.9%)	5 (2.1%)	6 (2.6%)	25 (10.7%)	1 (0.4%)		
	Total	63 (27%)	36 (15.5%)	36 (15.5%)	18 (7.7%)	75 (32.2%)	5 (2.1%)		
Weather	Agree	32 (13.7%)	19 (8.2%)	17 (7.3%)	7 (3%)	39 (16.7%)	4 (1.7%)	5.56	0.850
	Disagree	9 (3.9%)	6 (2.6%)	6 (2.6%)	2 (0.2%)	7 (3%)	0 (0.0%)		
	Uncertain	22 (9.4%)	11 (4.7%)	13 (5.6%)	9 (3.9%)	29 (12.4%)	1 (0.4%)		
	Total	63 (27%)	36 (15.5%)	36 (15.5%)	18 (7.7%)	75 (32.2%)	5 (2.1%)		
Atmospheric pressure	Agree	38 (16.3%)	19 (8,2%)	23 (9.9%)	10 (4.6%)	36 (15.5%)	4 (1.7%)	5.55	0.851
	Disagree	6 (2.6%)	5 (2.1%)	2 (0.9%)	2 (0.9%)	9 (3.9%)	0 (0.0%)		
	Uncertain	19 (8.2%)	12 (5.2%)	11 (4.7%)	6 (2.6%)	30 (12.9%)	1 (0.4%)		
	Total	63 (27%)	36 (15.5%)	36 (15.5%)	18 (7.7%)	75 (32.2%)	5 (2.1%)		
Hormonal changes	Agree	33 (14.2%)	22 (9.4%)	28 (12%)	12 (5.2%)	58 (24.9%)	4 (1.7%)	18.77	0.043*
	Disagree	2 (1.7%)	4 (1.7%)	2 (0.9%)	0 (0.0%)	3 (1.3%)	1 (0.4%)		
	Uncertain	26 (11.2%)	10 (4.3%)	6 (2.6%)	6 (2.6%)	14 (6%)	0 (0.0%)		
	Total	63 (27%)	36 (15.5%)	36 (15.5%)	18 (7.7%)	75 (32.2%)	5 (2.1%)		
Inadequate sleep	Agree	55 (23.6%)	27 (11.6%)	29 (12.4%)	14 (6%)	69 (29.6%)	5 (2.1%)	14.84	0.138
	Disagree	1 (0.4%)	4 (1.7%)	2 (0.9%)	3 (1.3%)	2 (0.9%)	0 (0.0%)		
	Uncertain	7 (3%)	5 (2.1%)	5 (2.1%)	1 (0.4%)	4 (1.7%)	0 (0.0%)		
	Total	63 (27%)	36 (15.5%)	36 (15.5%)	18 (7.7%)	75 (32.2%)	5 (2.1%)		
Foods and chemicals like MSG	Agree	24 (10.3%)	29 (8.2%)	18 (7.7%)	6 (2.6%)	34 (14.6%)	4 (1.7%)	6.71	0.752
	Disagree	9 (3.9%)	4 (1.7%)	4 (1.7%)	3 (1.3%)	7 (3%)	0 (0.0%)		
	Uncertain	20 (12.9%)	13 (5.6%)	14 (6%)	9 (3.9%)	34 (14.6%)	1 (0.4%)		
	Total	63 (27%)	36 (15.5%)	36 (15.5%)	18 (7.7%)	75 (32.2%)	5 (2.1%)		
High caffeine intake	Agree	35 (15%)	28 (12%)	24 (10.3%)	12 (5.2%)	55 (23.6%)	3 (1.3%)	8.10	0.619
	Disagree	10 (4.3%)	4 (1.7%)	4 (1.7%)	2 (0.9%)	6 (2.6%)	1 (0.4%)		
	Uncertain	18 (7.7%)	4 (1.7%)	8 (3.4%)	4 (1.7%)	14 (6%)	1 (0.4%)		
	Total	63 (27%)	36 (15.5%)	36 (15.5%)	18 (7.7%)	75 (32.2%)	5 (2.1%)		
Starvation	Agree	38 (16.3%)	22 (9.4%)	18 (7.7%)	9 (3.9%)	42(18%)	2 (0.9%)	4.34	0.930
	Disagree	6 (2.6%)	3 (1.3%)	4 (1.7%)	2 (0.9%)	12 (5.2%)	1 (0.4%)		
	Uncertain	19 (8.2%)	11 (4.7%)	14 (6%)	7 (3%)	21 (9%)	2 (0.9%)		
	Total	63 (27%)	36 (15.5%)	36 (15.5%)	18 (7.7%)	75 (32.2%)	5 (2.1%)		

The secondary analysis results showed a statistical significance between the participants’ years of medical schooling and their knowledge of treatment modalities for managing migraines (Table 5). Students in all years of their medical school training showed the highest agreement with medications as the preferred treatment option, followed by rest. Knowledge of medications as a treatment option for migraines was highest for fifth-year medical students, followed by first-year (21.9%) and second-year (8.2%) medical students (p ≤ 0.001). Knowledge of rest as a treatment option for migraines was highest for first-year medical students (16.7%), followed by second-year students (6.5%) (p ≤ 0.001). Very few students recognized alternative medicines, such as acupuncture, as options for managing migraines.

**Table 5:** Chi-square analysis between participant year of study and modalities of management of migraine Chi-square analysis. ** significance <0.00, Pearson's Chi-Sq value: 38.62. Results are displayed as number (%)

Managment	Year	1st	2nd	3rd	4th	5th	Intern	p-value
Modalities of managing migraines	Rest	39 (16.7%)	15 (6.4%)	14 (6%)	5 (2.1%)	21 (9%)	2 (0.9%)	<0.001**
	Medication	19 (8.2%)	13 (5.6%)	19 (8.2%)	11 (8.2%)	51 (21.9%)	3 (1.3%)	
	Routine physical activities	2 (0.9%)	5 (2.1%)	3 (1.3%)	0 (0.0%)	1 (0.4%)	0 (0.0%)	
	Alternative medicine	3 (1.3%)	3 (1.3%)	0 (0.0%)	2 (0.9%)	2 (0.9%)	0 (0.0%)	
	Total	63 (27%)	36 (15.5%)	36 (15.5%)	18 (7.7%)	75 (32.2%)	5 (2.1%)	

## Discussion

This multi-university cross-sectional study investigated medical students’ knowledge of migraine management and recognition of triggering factors. To the best of our knowledge, this is the first study of its kind to assess the knowledge and perception of migraine triggers and treatments among medical students in Saudi Arabia, more specifically in Riyadh. While other studies focus on trained professionals, this study focused on students and sheds light on changes that can be made in the curriculum/practical training to ensure that future doctors are adequately trained to identify and manage migraines [23]. Similar studies have also been conducted on nursing students [24].

Most students in the present study were male (81%) and either in their fifth year (32.2%) or first year (27%) of medical school. This gender discrepancy is unusual, as most research papers have reported a female predominance in their results [24-26]. The majority of the study participants (60%) indicated that they believed the age at presentation of migraine among most patients to be around 17-30 years. Other studies have also reported a similar age range at presentation [27].

The most recognized management method for migraines was medication (49.8%), followed by rest (41%). The least recognized method was alternative therapies (e.g., cupping or acupuncture) (4.3%). A chi-square analysis revealed this difference to be statistically significant. Similar results were reported in a study focusing on the medical student population in Saudi Arabia that suffered from migraines [28].

In the present study, the participants recognized exposure to light (90.6%), stress (86.7%), and inadequate sleep (85.4%) as the major factors triggering migraines. Only a limited number of studies have examined medical students’ perception knowledge of migraine-triggering factors; some studies have reported this in the context of medical students who suffer from migraines. This study has also demonstrated that the majority of students reported light, inadequate sleep, and emotional stress as some of the main triggering factors [28].

Interestingly, comparisons of the knowledge of triggering factors and years of medical training among the participants yielded statistically significant differences. The factors contributing to migraine risk that were statistically significant included knowledge of migraine presentation in female patients, certain odors, and hormonal influences. This finding is in agreement with that of other studies [23,24].

Limitations

This study is not without limitations. The gender discrepancy among the participants, as reflected in the predominance of male participants in this study, may indicate that the results do not accurately represent all medical students in Riyadh, as female medical students were underrepresented. Other confounding factors, such as students having experienced migraines themselves, were neither ruled out nor addressed. Finally, as this study was conducted in Riyadh, a city in Saudi Arabia, the results cannot be generalized to medical student populations outside this setting.

## Conclusions

This study demonstrated medical students’ knowledge and perceptions regarding migraine management and trigger factors. Although the students showed appropriate recognition of the treatment methods, further investigation of the types of treatments should be carried out in future studies. The students also identified the main triggering factors of migraines. Future studies may focus on investigating medical students’ knowledge of managing each known triggering factor.
